# Supplementary material for: Microinvasion by Streptococcus pneumoniae induces epithelial innate immunity during colonisation at the human mucosal surface
Source: Nat Commun. 2019 Jul 16;10:3060. doi: 10.1038/s41467-019-11005-2 (PMC6635362; doi:10.1038/s41467-019-11005-2)
Supplement: Supplementary file 1 — Supplementary Information [file 41467_2019_11005_MOESM1_ESM.pdf]

Microinvasion by *Streptococcus pneumoniae* induces epithelial innate immunity during colonisation at the human mucosal surface

Weight CM et al

SUPPLEMENTARY INFORMATION

SUPPLEMENTARY TABLES

Panel composition and specifications of the multiparametric flow cytometry panel used for the identification of the epithelial and immune cells from nasal cells, and for the epithelial activation markers from Detroit 562 cell lines.

Antibody, Cat No.	Company, clone	Antibody	µl per sample	Biolegend Isotype	Filter on LSRII	Voltage run on LSRII
TCRγδ – PE-CF594	BD, B1	Mouse/IgG1,k	5	n/a	610/20	500
IL22Ra1-PerCP	R&D	Mouse/IgG1	5	#400147	695/40	661
CD54 –BV711	MAH-HA58, BD	Mouse/IgG1,k	5	#400167	710/50	615
CD107a –BV650	Biolegend, H4A3	Mouse/IgG1,k	5	n/a	655/8	492
CD3-APC.Cy7	BD, SK7	Mouse/IgG1,k	3	n/a	780/60	710
CD45-PacOrange	Life technologies, HI30	Mouse / IgG1	3	n/a	525/50	532
CD4 –BV605	Biolegend, RPA-T4	Mouse IgG1,k	3	n/a	610/20	500
CD40-PE.Cy7	Biolegend, 5C3	Mouse/IgG1,k	3	#400125	780/60	693
CD218a-APC	eBiosciences, H44	Mouse/IgG1,k	5	n/a	660/20	542
EpCAM-PE	Biolegend, 9C4	Mouse/IgG2b,k	1.5	#401207	575/26	488
CD8 –BV785	Biolegend SK1	Mouse/IgG1,k	1.5	n/a	780/60	591
HLA-DR-FITC	Biolegend L243	Mouse/ IgG2a,k	0.75	#400207	530/30	400 or 500
IL-22RA1-PerCP	Bio Techne Ltd, 305405	Mouse/IgG1	5	#400147	695/40	551
IL-22RA1-PerCP	Bio Techne Ltd, 305405	Mouse/IgG1	5	#400147	710/50	551
CD54 –APC	Life Technologies 1H4	Mouse/IgG1,k	5	#555745	670/30	592
CD107a –PE	Life Technologies H4A3	Mouse/IgG1,k	5	#555749	582/15	510
CD40-BV421	Biolegend, 5C3	Mouse/IgG1,k	5	#562438	450/50	528
HLA-DR-BUV395	BD, G46-6	Mouse/ IgG2a,k	5	#563809	379/28	538

A multiparametric flow cytometry panel composed of twelve (*in vivo*) and five (*in vitro,* last five) different monoclonal antibodies was developed to analyze the epithelial activation and

immunological cell populations obtained from nasal cell samples by flow cytometry. Electronic compensation was set using CompBeads (BD Biosciences) and the FacsDiva automated compensation according to manufacturer's instructions.

Panel of Antibodies for Immunofluorescence.

**Antibody**	**Company, clone**
JAM-A	Santa Cruz, 1H2A9
Claudin 4	Life Technologies
β catenin	NEBiolabs Ltd, L54E2
Pneumococcal Antiserum Type 4	SSI Diagnostica (TIGR4)
Pneumococcal Antiserum Pool Q	SSI Diagnostica (6B and 23F)
Wheat Germ Agglutinin	Vector labs, Rhodamine conjugated
Goat anti-mouse	Life Technologies (AF- 546,647)
Goat anti rabbit	Life Technologies (AF-488.546,647)
Goat anti-mouse-HRP	Dako (P0047)

Samples were incubated with primary antibodies for one hour (1:100). After washing, samples were incubated with secondary antibodies (1:500) for 45 minutes.

SUPPLEMENTARY FIGURES

Supplementary Figure 1

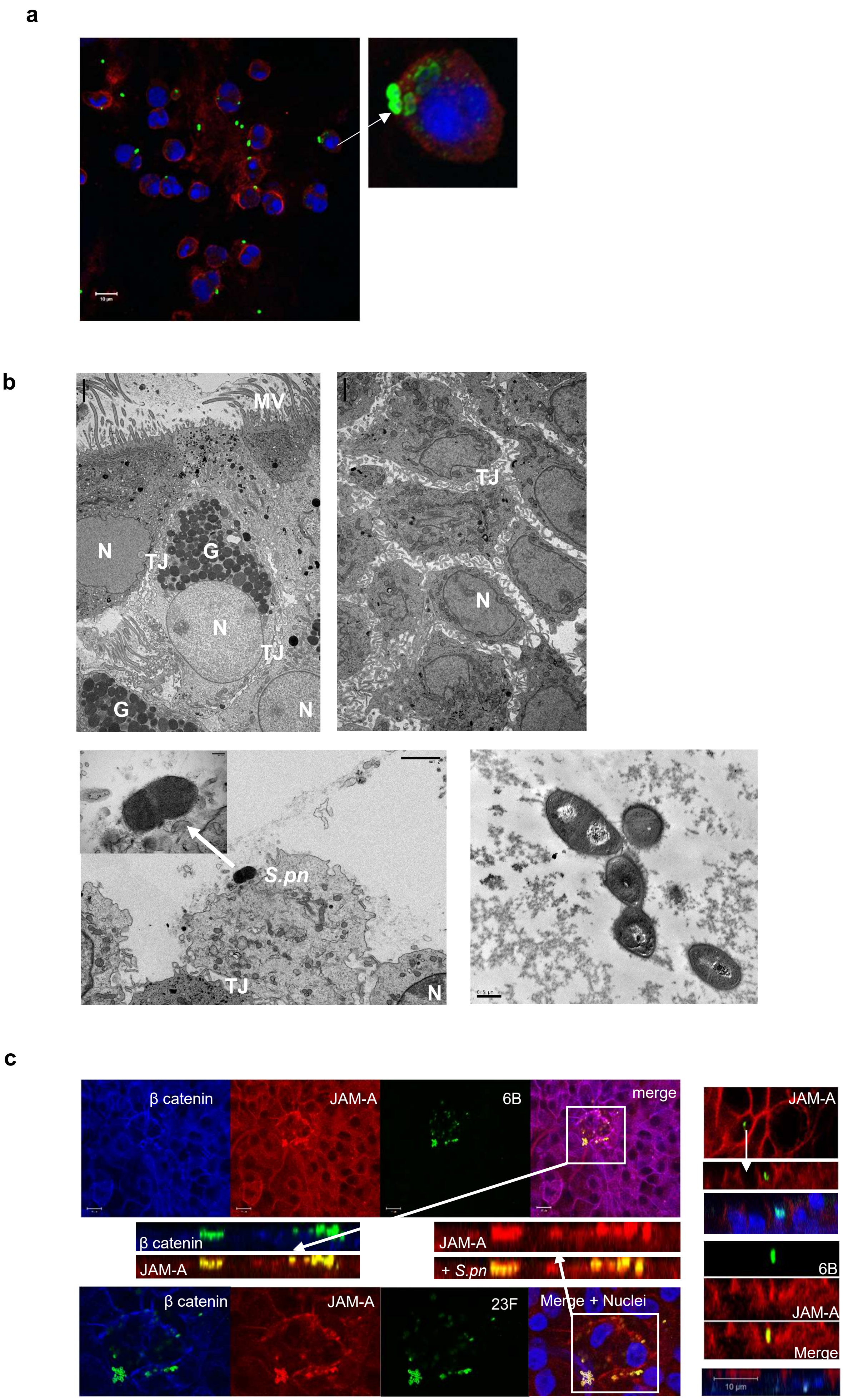

Supplementary Figure 1. *S. pneumoniae* colonisation of the human nasal mucosa is associated with adhesion, micro-colony formation and micro-invasion

(a) *In vivo* Immunofluorescence: Cells collected from nasal curette biopsies from the EHPC model were prepared for microscopy and stained for surface carbohydrates (red), 6B (green) and DAPI (blue). In addition to epithelial cells collected from nasal biopsies, immune cells were readily observed as indicated by round, multi-nuclei cells. (b) *In vivo* electron microscopy: cells collected from nasal curette biopsies were prepared for TEM. MV – microvilli, N – nucleus, TJ – tight junctions, G – goblet cell, *S.pn* – *Streptococcus pneumoniae.* (top left) epithelial cell architecture preserved, scale bar 2μm; (top right) intact sheet of cells with tight junctions visible, scale bar 2μm; (bottom left) a diplococci adhered to the cell surface, scale bar 2μm (insert 200nm); (bottom right) a pneumococcal chain, scale bar 0.5μm. (c) *Ex vivo* Immunofluorescence: primary cells were cultured for 30 days on an air-liquid interface and incubated with 1 million pneumococci (6B or 23F) for 3 hours. (left hand side) Cells were fixed and stained for β catenin (blue), JAM-A (red) and pneumococci capsule (green). Nuclei in blue. The corresponding XZ images are also shown. Other images represent (top right) intracellular, (middle) paracellular and (bottom right) basal localisation of *S. pneumoniae* within the epithelial monolayers. Images represent one experiment from one transwell insert per condition.

Supplementary Figure 2

a

Edit Save Matrix SSM

Show All	FITC-A :: HLAD...	PE-A :: Epcam, ...	PE-Texas Red-...	PerCP-Cy5-5-A...	PE-Cy7-A :: CD...	Pacific Blue-A :: ...	Pacific Orange-...	BV605-A :: CD4	BV650-A :: CD1...	BV711-A :: CD5...	BV786-A :: CD8	APC-A :: CD218...	Alexa Fluor 700-A	APC-Cy7-A :: C...
FITC-A :: HLADR, CD66b	100	13.8158	6.2607	1.2509	0.1647	0.0115	4.5169	0.2008	0.0386	0.0232	0.0057	0.0077	0	0.0113
PE-A :: Epcam, EpCam	1.5588	100	41.8032	10.9439	1.3986	0	0.1784	2.568	0.5518	0.2166	0.0608	0.0068	0	0
PE-Texas Red-A :: Tgd, TCRgd, ...	0.258	8.7632	100	39.5663	5.9608	0.0078	0.0486	6.2893	1.4458	0.7501	0.3323	0.2284	0.07	0.0725
PerCP-Cy5-5-A :: IL22RA, IL22Ra...	0	0.0132	0.0151	100	15	0	0.0364	0.0099	1	7	4.8525	3.5	5.7058	4.5119
PE-Cy7-A :: CD40, HLA-DR	0.1204	0.6791	0.3295	0.2717	100	0	0.0193	0.0211	1	0.0088	3.1261	1	0.3884	6.7784
Pacific Blue-A :: Viability, Live D...	0.6054	0.0625	0.0292	0	0.017	100	153.2052	3.185	0.6535	0.2589	0.14	0.0785	0	0
Pacific Orange-A :: CD45	0.8344	0.4321	0.3725	0.149	0	0.3069	100	25.8392	7.7307	3.4337	1.4771	0.0633	0	0.0067
BV605-A :: CD4	0.0085	1.136	13.6955	6.3801	1.3388	1.0337	0.8962	100	36.7231	15.2	7.178	0.3013	0.0927	0.0419
BV650-A :: CD107a, CD107	0.0836	0.0767	0.5133	4.4226	0.7271	1.9928	1.5505	15.7727	100	43	16.5365	31.6808	10.4587	5.4768
BV711-A :: CD54, PD1	0.0485	0.0388	0.0194	34.9144	13	3.2091	2.616	0.1562	0.6688	100	77.1057	0.5443	43.6319	30.3307
BV786-A :: CD8	0.0827	0.0489	0.0414	0.1091	13.5479	2.5174	3.0766	0.3293	0.2572	0.5812	100	0.0897	0.8839	10.8286
APC-A :: CD218a, CD16	0.0083	0.0058	0.0219	1.3207	0.2895	0.0152	0.0417	0.0381	3.7194	1.25	0.734	100	30.8557	14.8498
Alexa Fluor 700-A	0	0	0	0	0	0	0	0	0	0	0	0	100	0
APC-Cy7-A :: CD3	0.0352	0.0294	0.0176	0.0529	2.3721	0	0	0	0.0455	0.0303	3.6438	0.8585	3.4813	100

b

	Baseline	Day 2	Day 6	Day 9	Day 14-29
Carriage negative	(5)(9)(14)	(3)(4)(11)	(3)(7)(17)	(3)(5)(15)	(1)(5)(15)
Carriage positive	(8)(10)(13)	(9)(13)(18)	(4)(9)(14)	(6)(11)(17)	(5)(8)(11)

c

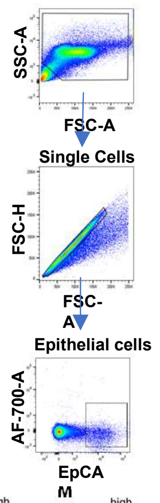

d

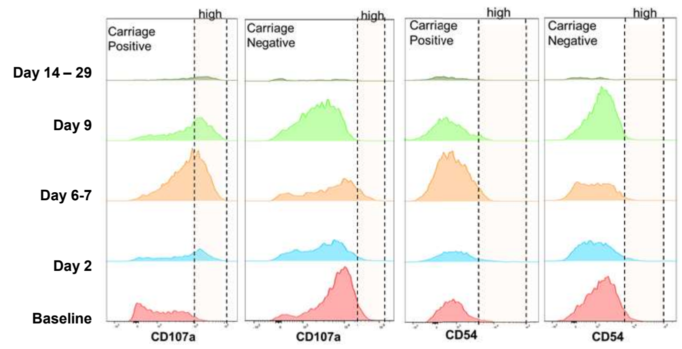

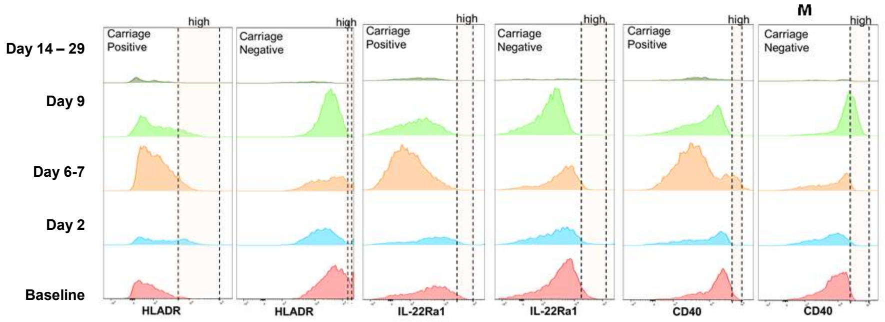

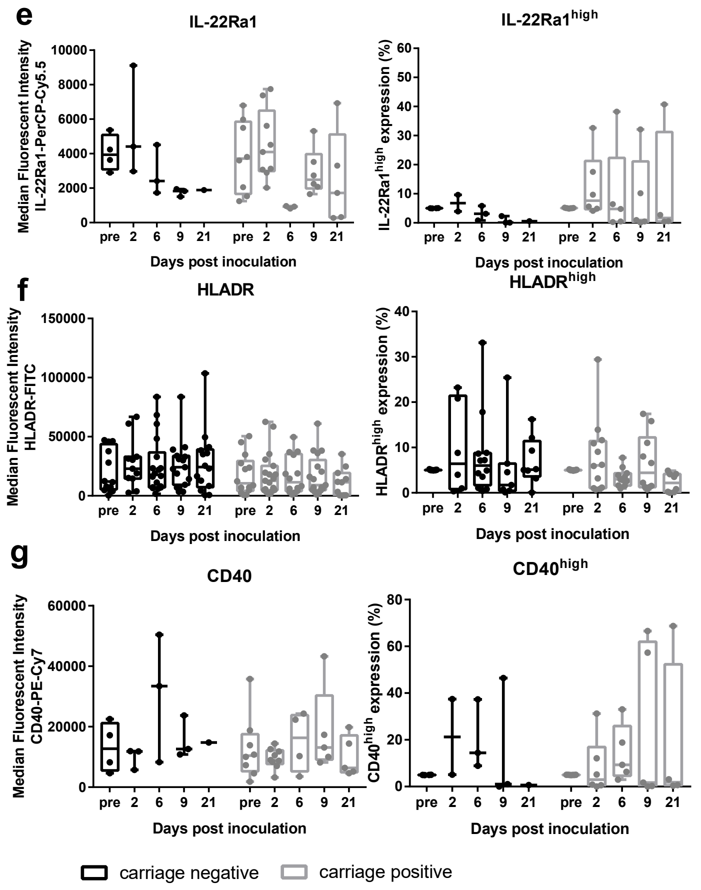

Supplementary Figure 2. Epithelial surface marker expression in response to *S. pneumoniae in vivo*

(a-d) *In vivo* FACS: Cells collected from nasal scrapes were prepared for Flow Cytometry Analysis. (a) Compensation matrix for flow cytometry panel. (b) Sample sizes for each day and carriage status. Sample sizes refer to: (CD40, CD54, IL-22Ra1) (CD107a) (HLADR) respectively. (c) Samples were gated into 'all cells', 'single cells', and finally 'EpCAM positive' cells, against an empty channel (AF-700-A) to analyse the epithelial cell population of the samples. Representative plots are shown. (d) An example of histograms for each epithelial activation marker, from a carriage positive and carriage negative volunteer. From these histograms, the cells expressing the highest 5% of activation markers, from baseline, were gated (high), to generate comparisons between sampling days within volunteers. Median fluorescence intensity and high surface marker-expressing cells (≥95% of the baseline expression) for epithelial surface expression of (e) IL-22Ra, (f) HLADR and (g) CD40. Results are from a minimum of two volunteers. Black circles show carriage negative, and grey circles show carriage positive, volunteers. Boxplots represent the median (centre line) and interquartile range (box), minimums and maximums (whiskers).

Supplementary Figure 3

a **Detroit 562 cells**

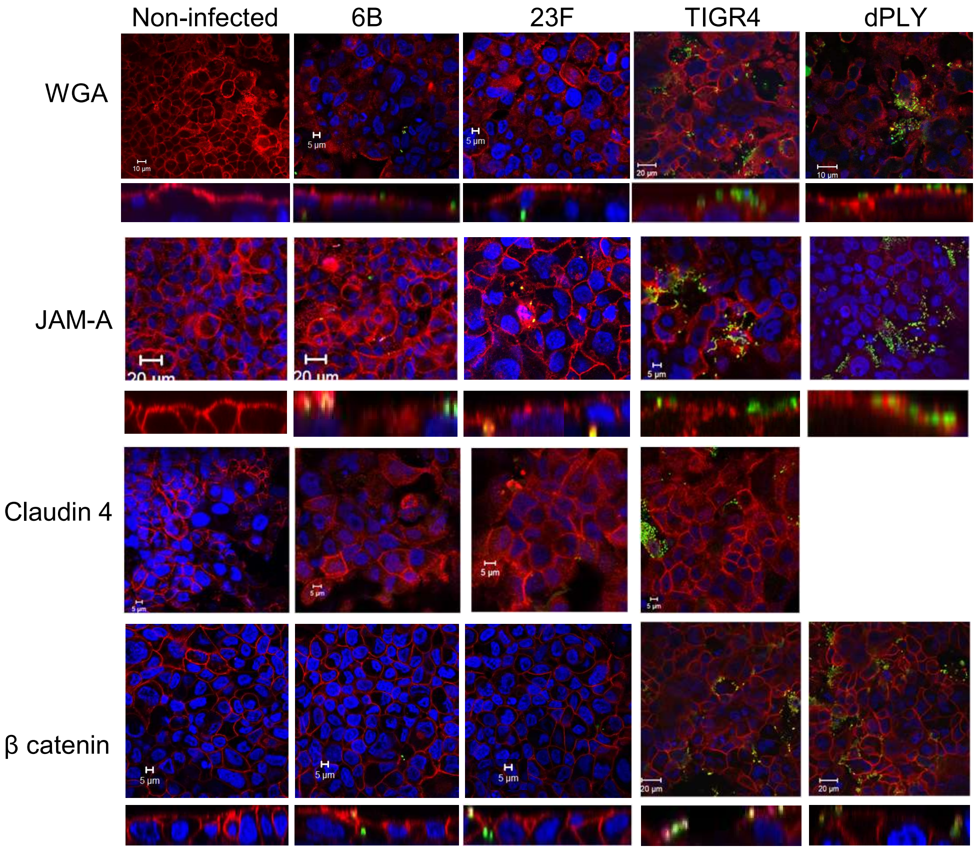

b

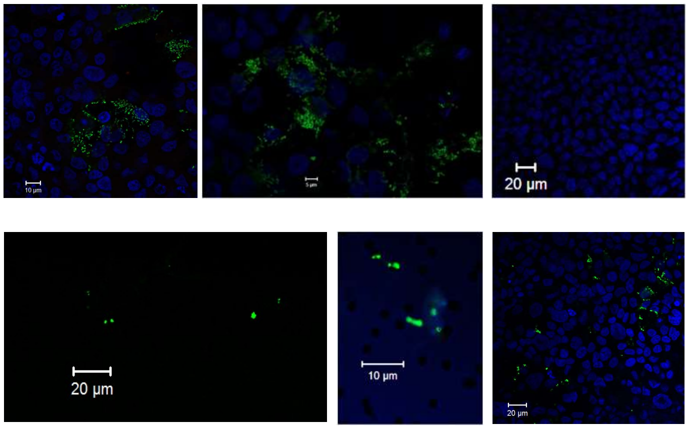

Supplementary Figure 3. Junction association of *S. pneumoniae* in Detroit 562 cells

(a) Detroit 562 cells were stained for *S. pneumoniae* (green) and either WGA, JAM-A, Claudin 4 or β catenin (red) three hours post infection. Nuclei are in blue. Representative XY images and XZ stacks of cells are shown for examples of surface 6B; basal located 23F; micro-colony of TIGR4, surface bound dPly. N = 3 with replicates. (b) Negative controls for immunofluorescence (top left) JAM-A (ms) primary antibody and goat-anti rabbit secondary with TIGR4-FAMSE. (top middle) TIGR4-FAMSE and goat-anti mouse secondary. (top right) antiserum and goat anti-mouse secondary. (bottom left) blank insert with FAMSE-23F with goat anti-mouse and goat anti-rabbit secondary. (bottom middle) Blank insert with JAM-A and goat anti-mouse secondary. (bottom right) FAMSE-TIGR4 with JAM-A antibody.

Supplementary Figure 4 A549 cells

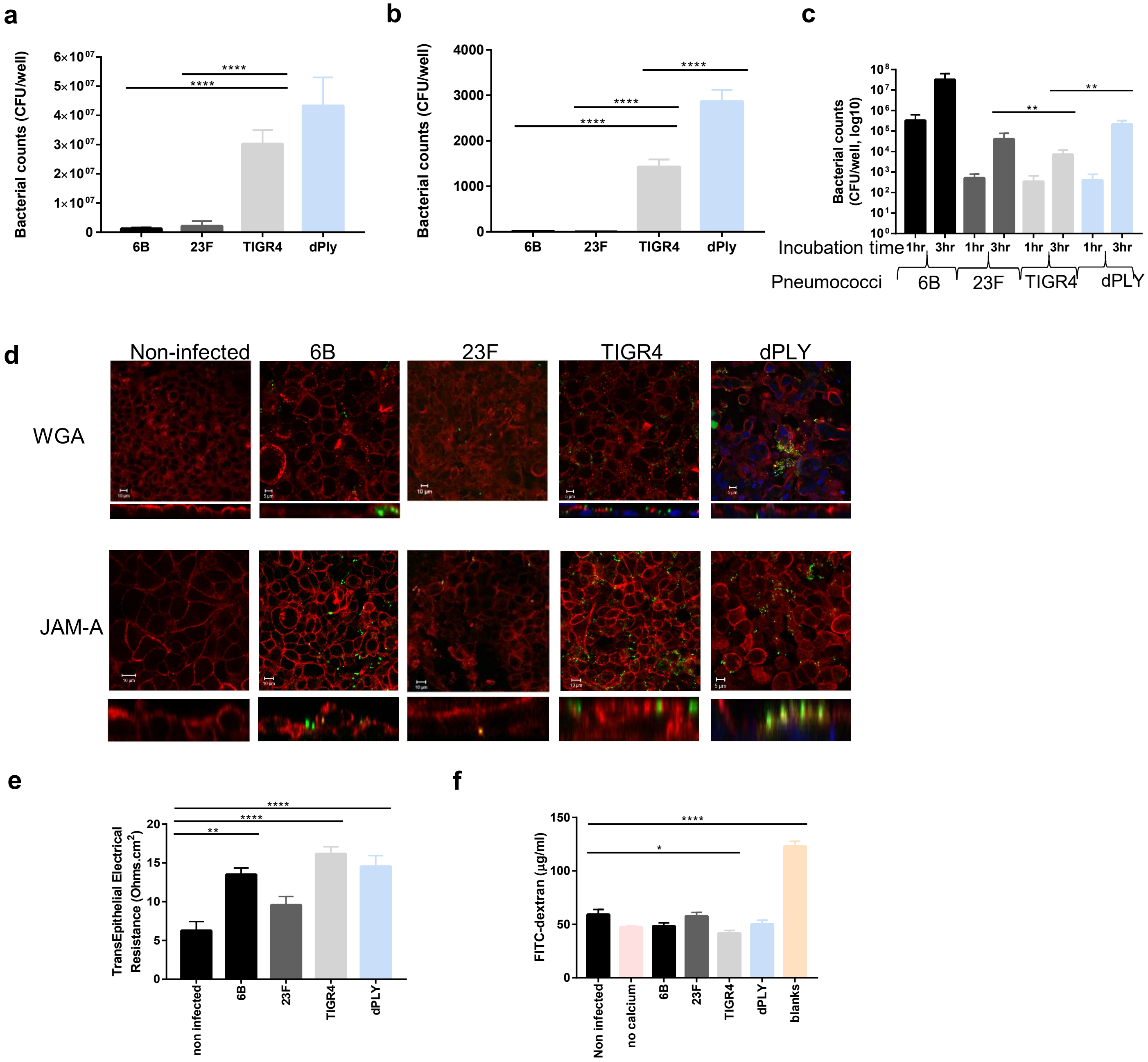

Calu3 cells

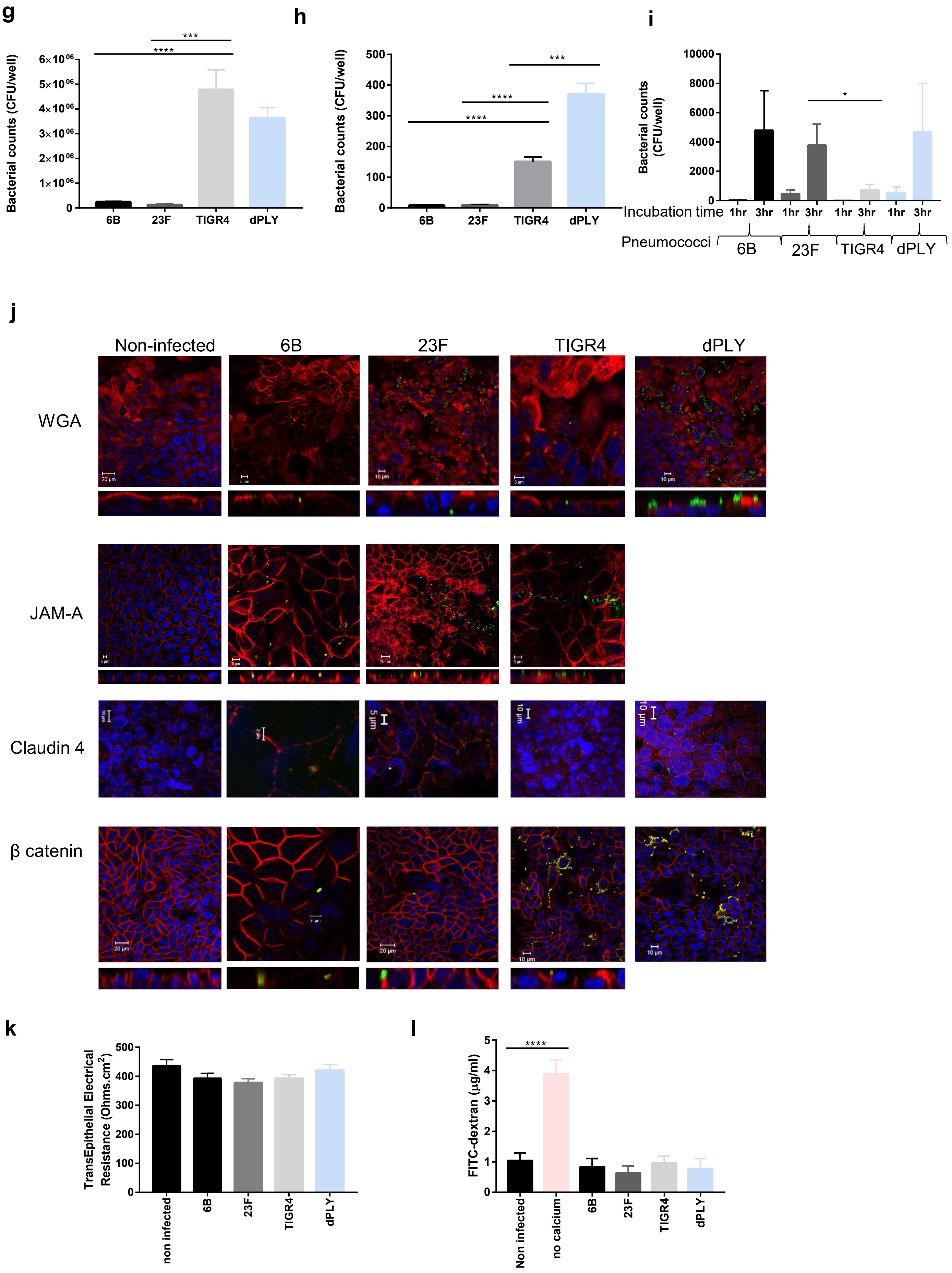
g
Bacterial counts (CFU/well)
6×10^06
5×10^06
4×10^06
3×10^06
2×10^06
1×10^06
0
***
****
6B
23F
TIGR4
dPLY
h
Bacterial counts (CFU/well)
500
400
300
200
100
0
***
****
****
6B
23F
TIGR4
dPLY
i
Bacterial counts (CFU/well)
10000
8000
6000
4000
2000
0
*
Incubation time 1hr 3hr 1hr 3hr 1hr 3hr 1hr 3hr
Pneumococci 6B 23F TIGR4 dPLY
j
Non-infected
6B
23F
TIGR4
dPLY
WGA
JAM-A
Claudin 4
β catenin
k
TransEpithelial Electrical Resistance (Ohms.cm2)
500
400
300
200
100
0
non infected
6B
23F
TIGR4
dPLY
l
FITC-dextran (µg/ml)
5
4
3
2
1
0
****
Non infected
no calcium
6B
23F
TIGR4
dPLY

Supplementary Figure 4. Epithelial adherence, endocytosis and transmigration by *S. pneumoniae* varies by pneumococcal strain and is modulated by pneumolysin, without affecting barrier integrity.

(a - f) A549 cells were stimulated with *S. pneumoniae* for three hours and CFU measured for (a) association, (b) internalisation and (c) transmigration over time. ****P = <0.0001 for adhesion and invasion assays; **P = 0.0049 at three hours transmigration for 23F v TIGR4 and TIGR4 v dPLY. N = 7. (d) Cells stained for *S. pneumoniae* (green) and either WGA, JAM-A, Claudin 4 or β catenin (red). XY and XZ images represent associations and destinations of the different pneumococcal strains with different host proteins. (e – f) Barrier function was assessed before and after three hours exposure to *S. pneumoniae.* (e) There were no significant differences in TEER at the start of the experiment. N = 4. ****P = <0.0001. (f) Dextran leakage was recorded. Blank inserts = positive control (****P < 0.0001). *P = 0.0108 non-infected v TIGR4. n = 5.

(g - l) Calu3 cells were stimulated with *S. pneumoniae* for three hours and CFU measured for (g) association, (h) internalisation and (i) transmigration over time. ****P = <0.0001 for adhesion and invasion. (i) Transmigration at three hours infection between 23F v TIGR4 *P = 0.0281. N = 4. (j) Calu3 cells stained for *S. pneumoniae* and either WGA, JAM-A, Claudin 4 or β catenin. XY and XZ images represent associations and destinations of the different pneumococcal strains with different host proteins. (k - l) Barrier function was assessed during exposure to *S. pneumoniae.* (k) TransEpithelial Electrical Resistance (TEER) was recorded before and after the experiment: n = 7. TEER was not significantly different between inserts before or after pneumococci were added (P = 0.7507 and 0.1088 respectively). (l) Dextran leakage was measured and calcium withdrawal = positive control (****P = <0.0001, n = 3, Unpaired T-Test). No significant differences were detected in permeability with pneumococcal infection, P = 0.0560. N = 5. Error bars represent standard error of the mean.

Supplementary Figure 5

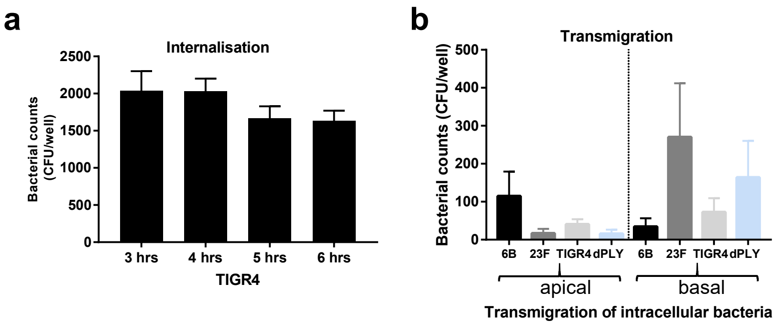

Supplementary Figure 5. Internalised pneumococci egress from the epithelium

(a) Detroit 562 cells were incubated with *S. pneumoniae* for three hours, washed, treated with gentamicin for 1 hour, washed and the cultures incubated for further time points to measure (a) bacterial internalisation. N = 4. (b) Bacteria that were released into the apical or basal chamber after one hour were then counted. N = 3. Similar results were also observed with Calu 3 cells (data not shown). Error bars represent standard error of the mean.

Supplementary Figure 6

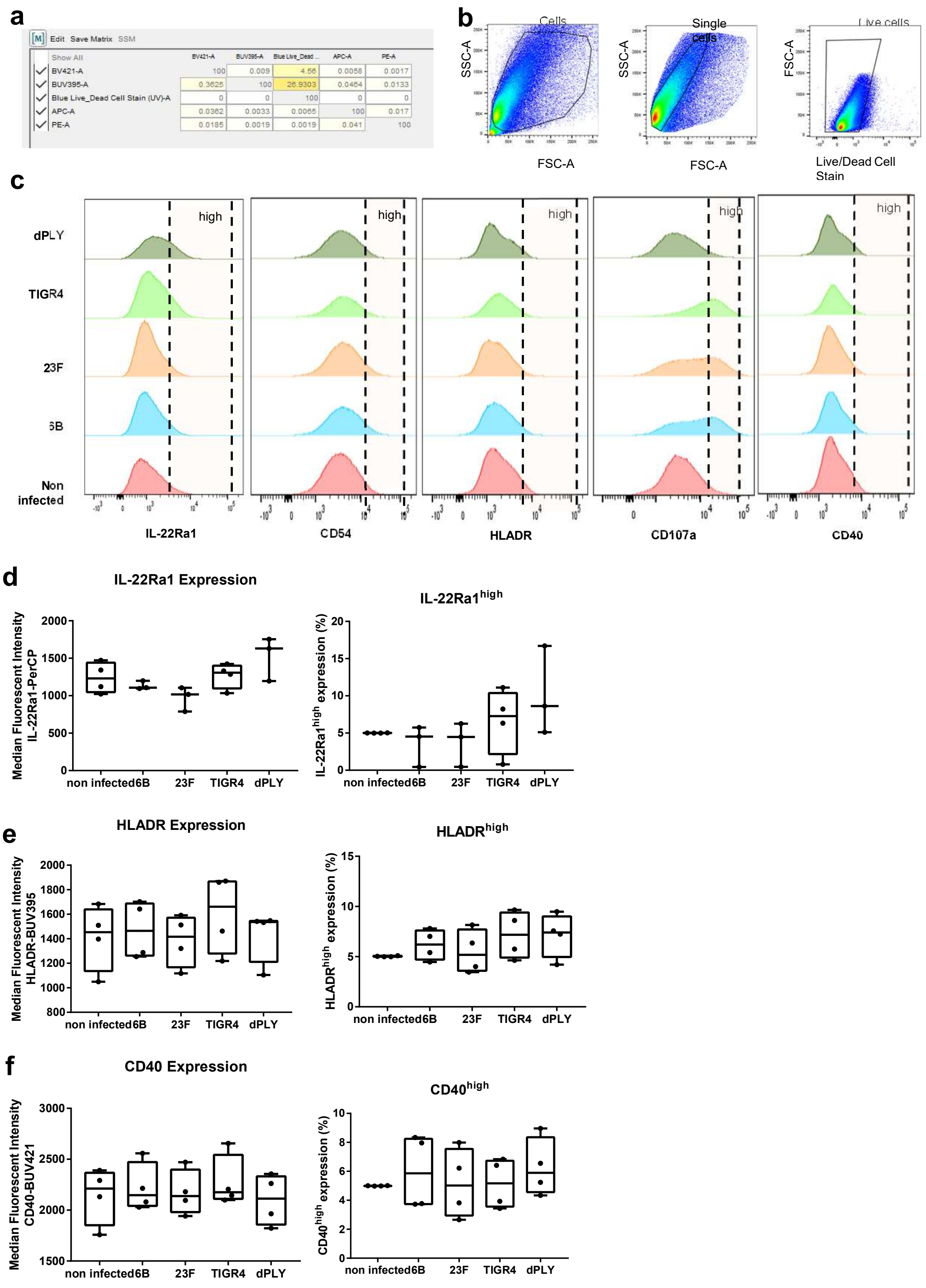

Supplementary Figure 6. Epithelial surface marker expression in response to *S. pneumoniae in vitro*

(a - c) *In vitro* FACS: Cells collected from Detroit 562 monolayers (compensation matrix shown in (a)) were gated into ‘all cells’ (b), the population further defined to ‘single cells’, and finally exclusion of dead cells, lead to the live cell population used for analyses. (c) Representative histograms for each epithelial activation marker are shown. Median fluorescence intensity and cells expressing the highest 5% of surface expression for (d) IL-22Ra, (e) HLADR and (f) CD40 were gated to generate comparisons between strains of pneumococci and non-infected cells. Boxplots represent the median (centre line) and interquartile range (box), minimums and maximums (whiskers).

Supplementary Figure 7

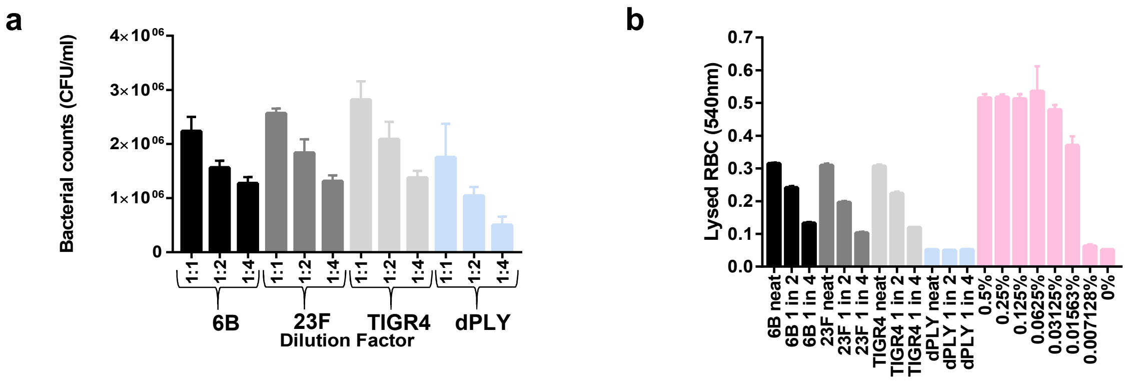

Supplementary Figure 7. Pneumolysin activity is comparable between pneumococcal strains.

(a - c) Bacterial preparations in phenol free RPMI (Invitrogen) were added to a solution of 2% red blood cells (EO labs) in a U-bottom 96 well plate for 30 minutes at 37°C / 5% CO_2. There were no significant differences between pneumococcal strain density for each dilution. (b) Serial dilutions of 0.5% saponin was used as a positive control for cell lysis. The plate was centrifuged for 1 minute at 1000g to pellet the un-lysed red blood cells and supernatant absorbance was read at 540nm. (c) There were no differences in lysis potential between the pneumococcal strains. dPly-TIGR4 mutant was used as a negative control for lysis. Error bars represent standard error of the mean.

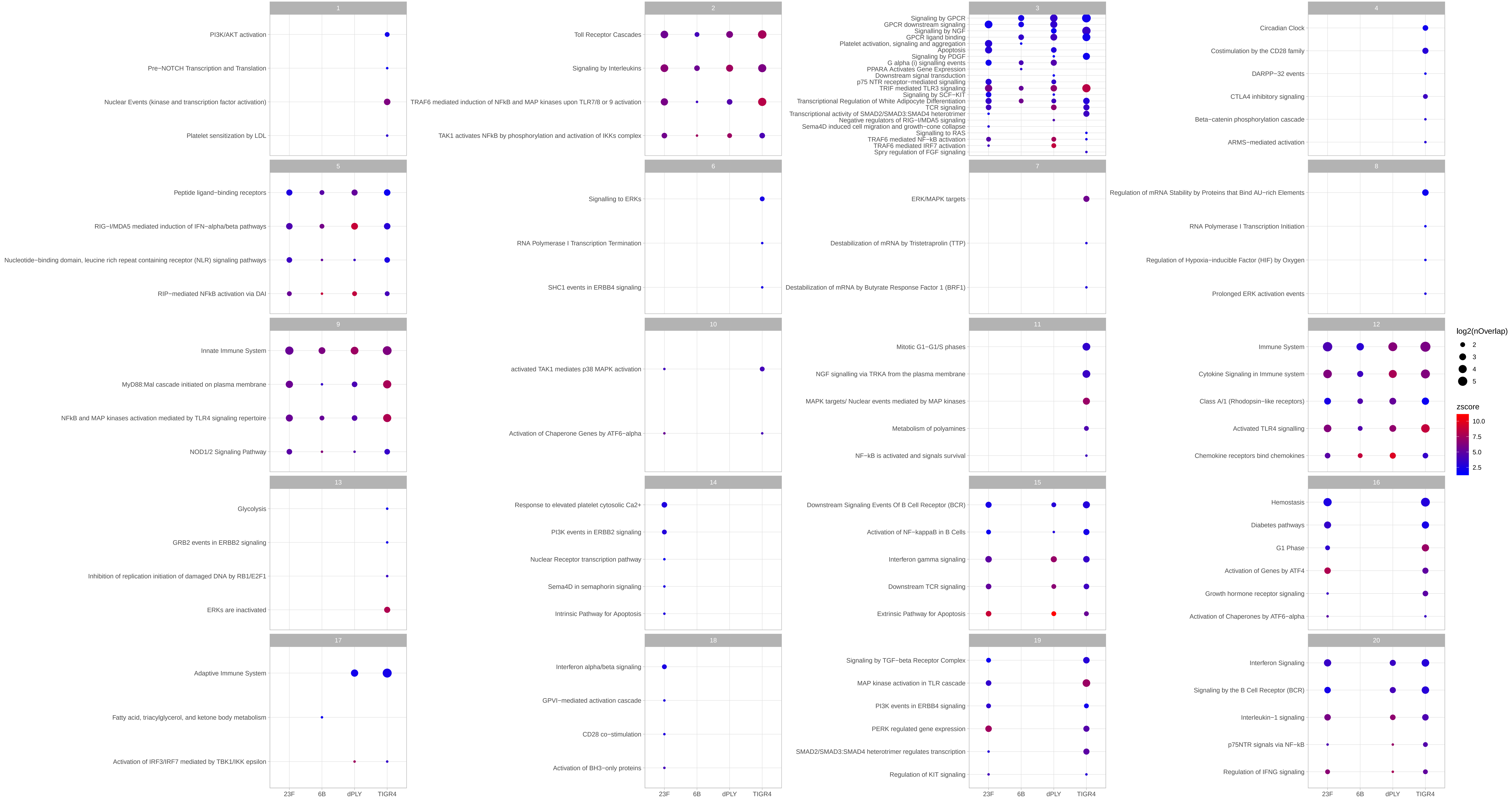
1
PI3K/AKT activation
Pre−NOTCH Transcription and Translation
Nuclear Events (kinase and transcription factor activation)
Platelet sensitization by LDL
2
Toll Receptor Cascades
Signaling by Interleukins
TRAF6 mediated induction of NFkB and MAP kinases upon TLR7/8 or 9 activation
TAK1 activates NFkB by phosphorylation and activation of IKKs complex
3
Signaling by GPCR
GPCR downstream signaling
Signaling by NGF
GPCR ligand binding
Platelet activation, signaling and aggregation
Apoptosis
Signaling by PDGF
G alpha (i) signalling events
PPARA Activates Gene Expression
Downstream signal transduction
p75 NTR receptor−mediated signalling
TRIF mediated TLR3 signaling
Signaling by SCF−KIT
Transcriptional Regulation of White Adipocyte Differentiation
TCR signaling
Transcriptional activity of SMAD2/SMAD3:SMAD4 heterotrimer
Negative regulators of RIG−I/MDA5 signaling
Sema4D induced cell migration and growth−cone collapse
Signaling to RAS
TRAF6 mediated NF−kB activation
TRAF6 mediated IRF7 activation
Spry regulation of FGF signaling
4
Circadian Clock
Costimulation by the CD28 family
DARPP−32 events
CTLA4 inhibitory signaling
Beta−catenin phosphorylation cascade
ARMS−mediated activation
5
Peptide ligand−binding receptors
RIG−I/MDA5 mediated induction of IFN−alpha/beta pathways
Nucleotide−binding domain, leucine rich repeat containing receptor (NLR) signaling pathways
RIP−mediated NFkB activation via DAI
6
Signalling to ERKs
RNA Polymerase I Transcription Termination
SHC1 events in ERBB4 signaling
7
ERK/MAPK targets
Destabilization of mRNA by Tristetraprolin (TTP)
Destabilization of mRNA by Butyrate Response Factor 1 (BRF1)
8
Regulation of mRNA Stability by Proteins that Bind AU−rich Elements
RNA Polymerase I Transcription Initiation
Regulation of Hypoxia−inducible Factor (HIF) by Oxygen
Prolonged ERK activation events
9
Innate Immune System
MyD88:Mal cascade initiated on plasma membrane
NFkB and MAP kinases activation mediated by TLR4 signaling repertoire
NOD1/2 Signaling Pathway
10
activated TAK1 mediates p38 MAPK activation
Activation of Chaperone Genes by ATF6−alpha
11
Mitotic G1−G1/S phases
NGF signalling via TRKA from the plasma membrane
MAPK targets/ Nuclear events mediated by MAP kinases
Metabolism of polyamines
NF−kB is activated and signals survival
12
Immune System
Cytokine Signaling in Immune system
Class A/1 (Rhodopsin−like receptors)
Activated TLR4 signalling
Chemokine receptors bind chemokines
13
Glycolysis
GRB2 events in ERBB2 signaling
Inhibition of replication initiation of damaged DNA by RB1/E2F1
ERKs are inactivated
14
Response to elevated platelet cytosolic Ca2+
PI3K events in ERBB2 signaling
Nuclear Receptor transcription pathway
Sema4D in semaphorin signaling
Intrinsic Pathway for Apoptosis
15
Downstream Signaling Events Of B Cell Receptor (BCR)
Activation of NF−kappaB in B Cells
Interferon gamma signaling
Downstream TCR signaling
Extrinsic Pathway for Apoptosis
16
Hemostasis
Diabetes pathways
G1 Phase
Activation of Genes by ATF4
Growth hormone receptor signaling
Activation of Chaperones by ATF6−alpha
17
Adaptive Immune System
Fatty acid, triacylglycerol, and ketone body metabolism
Activation of IRF3/IRF7 mediated by TBK1/IKK epsilon
18
Interferon alpha/beta signaling
GPVI−mediated activation cascade
CD28 co−stimulation
Activation of BH3−only proteins
19
Signaling by TGF−beta Receptor Complex
MAP kinase activation in TLR cascade
PI3K events in ERBB4 signaling
PERK regulated gene expression
SMAD2/SMAD3:SMAD4 heterotrimer regulates transcription
Regulation of KIT signaling
20
Interferon Signaling
Signaling by the B Cell Receptor (BCR)
Interleukin−1 signaling
p75NTR signals via NF−kB
Regulation of IFNG signaling
23F
6B
dPLY
TIGR4
log2(nOverlap)
2
3
4
5
zscore
10.0
7.5
5.0
2.5

Supplementary Figure 8. Epithelial transcriptomic responses to *S. pneumoniae in vitro*

All 20 individual pathways represented in the clusters from Figure 6c.
