# Supplementary material for: Microinvasion by Streptococcus pneumoniae induces epithelial innate immunity during colonisation at the human mucosal surface
Source: Nat Commun. 2019 Jul 16;10:3060. doi: 10.1038/s41467-019-11005-2 (PMC6635362; doi:10.1038/s41467-019-11005-2)
Supplement: Supplementary file 3 — Description of Additional Supplementary Files [file 41467_2019_11005_MOESM3_ESM.pdf]

Description of Additional Supplementary Files

File name: Supplementary Data 1
Description: Differentially upregulated genes from Detroit 562 cells infected with pneumococci; comparisons against non-infected cells.

File name: Supplementary Data 2
Description: Reactome Pathways and Interactome Genes from Detroit 562 cells infected with pneumococci.

File name: Supplementary Data 3
Description: Transcription factor binding site enrichment analysis.

File name: Supplementary Data 4
Description: Differential gene expression from the EHPC model.
