# Supplementary material for: Microinvasion by Streptococcus pneumoniae induces epithelial innate immunity during colonisation at the human mucosal surface
Source: Nat Commun. 2019 Jul 16;10:3060. doi: 10.1038/s41467-019-11005-2 (PMC6635362; doi:10.1038/s41467-019-11005-2)
Supplement: Supplementary file 8 — Reporting Summary [file 41467_2019_11005_MOESM8_ESM.pdf]

nature research

Corresponding author(s): Caroline Weight

Reporting Summary

Nature Research wishes to improve the reproducibility of the work that we publish. This form provides structure for consistency and transparency in reporting. For further information on Nature Research policies, see Authors & Referees and the Editorial Policy Checklist.

Statistical parameters

When statistical analyses are reported, confirm that the following items are present in the relevant location (e.g. figure legend, table legend, main text, or Methods section).

n/a	Confirmed	
☐	☒	The exact sample size (*n*) for each experimental group/condition, given as a discrete number and unit of measurement
☐	☒	An indication of whether measurements were taken from distinct samples or whether the same sample was measured repeatedly
☐	☒	The statistical test(s) used AND whether they are one- or two-sided<br>*Only common tests should be described solely by name; describe more complex techniques in the Methods section.*
☐	☒	A description of all covariates tested
☐	☒	A description of any assumptions or corrections, such as tests of normality and adjustment for multiple comparisons
☐	☒	A full description of the statistics including central tendency (e.g. means) or other basic estimates (e.g. regression coefficient) AND variation (e.g. standard deviation) or associated estimates of uncertainty (e.g. confidence intervals)
☒	☐	For null hypothesis testing, the test statistic (e.g. *F*, *t*, *r*) with confidence intervals, effect sizes, degrees of freedom and *P* value noted<br>*Give P values as exact values whenever suitable.*
☒	☐	For Bayesian analysis, information on the choice of priors and Markov chain Monte Carlo settings
☒	☐	For hierarchical and complex designs, identification of the appropriate level for tests and full reporting of outcomes
☒	☐	Estimates of effect sizes (e.g. Cohen's *d*, Pearson's *r*), indicating how they were calculated
☐	☒	Clearly defined error bars<br>*State explicitly what error bars represent (e.g. SD, SE, CI)*

Our web collection on statistics for biologists may be useful.

Software and code

Policy information about availability of computer code

Data collection	-Zen Zeiss software<br>-TissueFAXS software<br>-LSR2 Flow Cytometer
Data analysis	-Statistical analysis; GraphPrism v7<br>-Microscopy Images; LSM Image Browser<br>-Flow Cytometry; FlowJo<br>-RNASeq data; processing and analysis was conducted in R, language and environment for statistical computing (https://www.R-project.org). Mpping and generation of read counts per transcript were performed using Kallisto60, based on pseudoalignment. R/ Bioconductor package Tximport was used to import the mapped counts data and summarise the transcripts-level data into gene level data61. DESeq2 and SARTools packages62, were used for differential gene expression analysis.

For manuscripts utilizing custom algorithms or software that are central to the research but not yet described in published literature, software must be made available to editors/reviewers upon request. We strongly encourage code deposition in a community repository (e.g. GitHub). See the Nature Research guidelines for submitting code & software for further information.

Data

Policy information about availability of data

All manuscripts must include a data availability statement. This statement should provide the following information, where applicable:

- Accession codes, unique identifiers, or web links for publicly available datasets
- A list of figures that have associated raw data
- A description of any restrictions on data availability

RNAseq data for the EHPC model is referenced as GEO Series Number GSE124949. RNAseq data for the cell line is deposited in the ArrayExpress database at EMBL-EBI under accession number E-MTAB-7841. RNA processing codes used are found at https://github.com/cristina86cristina. Source data are provided as Source Data files.

Field-specific reporting

Please select the best fit for your research. If you are not sure, read the appropriate sections before making your selection.

☒ Life sciences ☐ Behavioural & social sciences ☐ Ecological, evolutionary & environmental sciences

For a reference copy of the document with all sections, see nature.com/authors/policies/ReportingSummary-flat.pdf

Life sciences study design

All studies must disclose on these points even when the disclosure is negative.

Sample size	Sample size on in vivo data was dependent on volunteers within the study (up to 18). Exact numbers are defined in the main table (microscopy and microbiology) and supplementary figure legends (flow cytometry). Sample sizes for in vitro experiments was a minimum of three independent experiments with technical replicates.
Data exclusions	For flow cytometry EHPC data, samples were excluded from analyses if the epithelial cell population was less than 500. For other exclusions, more than 2 standard deviations away from the mean were excluded (for Figure 3c: 1x 23F, 1x dPly for IL-8, 1x for dPly ICAM).
Replication	In vitro experiments were conducted in three or more independent experiments with technical replicates, unless stated otherwise. For EHPC data, entire samples were utilized for each assay.
Randomization	No randomization was required since experimental groups for the EHPC was double-blinded.
Blinding	For EHPC data, CMW was blinded for the entirety of the data collection as to the microbiology carriage status of each volunteer. FlowJo data collection was also blinded so that no bias was included in the gating strategy for the EHPC data.

Reporting for specific materials, systems and methods

Materials & experimental systems

n/a	Involved in the study	
☒	☐	Unique biological materials
☐	☒	Antibodies
☐	☒	Eukaryotic cell lines
☒	☐	Palaeontology
☒	☐	Animals and other organisms
☐	☒	Human research participants

Methods

n/a	Involved in the study	
☐	☐	ChIP-seq
☐	☒	Flow cytometry
☐	☐	MRI-based neuroimaging

Antibodies

Antibodies used

TCRγδ – PE-CF594 BD, B1 Mouse/IgG1,k
IL22Ra1-PerCP R&D Mouse/IgG1 #400147
CD54 –BV711 MAH-HA58, BD Mouse/IgG1,k isotype #400167
CD107a –BV650 Biolegend, H4A3 Mouse/IgG1,k
CD3-APC.Cy7 BD, SK7 Mouse/IgG1,k
CD45-PacOrange Life technologies, HI30 Mouse / IgG1
CD4 –BV605 Biolegend, RPA-T4 Mouse IgG1,k

CD40-PE.Cy7 Biolegend, 5C3 Mouse/IgG1,k, isotype #400125
CD218a-APC eBiosciences, H44 Mouse/IgG1,k
EpCAM-PE Biolegend, 9C4 Mouse/IgG2b,k, isotype #401207
CD8 –BV785 Biolegend SK1 Mouse/IgG1,k
HLA-DR-FITC Biolegend L243 Mouse/ IgG2a,k, isotype #400207
IL-22RA1-PerCP Bio Techne Ltd, 305405, Mouse IgG1, isotype #400147
CD54 –APC Life Technologies 1H4 Mouse/IgG1,k 5 #555745
CD107a –PE Life Technologies H4A3 Mouse/IgG1,k 5 #555749
CD40-BV421 Biolegend, 5C3 Mouse/IgG1,k 5 #562438
HLA-DR-BUV395 BD, G46-6 Mouse/ IgG2a,k 5 #563809
JAM-A Santa Cruz, 1H2A9
Claudin 4 Life Technologies
β catenin NEBiolabs Ltd, L54E2
Pneumococcal Antiserum Type 4 SSI Diagnostics, Oxford Biosystems Ltd (TIGR4)
Pneumococcal Antiserum Pool Q SSI Diagnostics, Oxford Biosystems Ltd (6B and 23F)
Wheat Germ Agglutinin Vector labs, Rhodamine conjugated
Goat anti-mouse Life Technologies (AF- 546,647)
Goat anti rabbit Life Technologies (AF-488.546,647)
Goat anti-mouse-HRP Dako, (P0047)

Validation

Compensation, Isotype controls and FL-1 controls were conducted for Flow Cytometry antibodies. No-primary controls were conducted for Immunofluorescence. Concentration of antibodies was chosen according to manufacturer instructions. Examples of all controls are included in the supplementary information.

Eukaryotic cell lines

Policy information about cell lines

Cell line source(s)

Human pharyngeal carcinoma Detroit 562 epithelial cells (ATCC_CCL-138). Human bronchial carcinoma Calu3 epithelial cells (ATCC_HTB-55). Human alveolar epithelial carcinoma A549 epithelial cells (ATCC_CCL-185)

Authentication

Purchased directly from ATCC so no personal authentication was conducted.

Mycoplasma contamination

All cell lines were tested for mycoplasma by PCR on a monthly basis and were continuously negative.

Commonly misidentified lines
(See ICLAC register)

N/A

Human research participants

Policy information about studies involving human research participants

Population characteristics

Following written informed consent, healthy non-smoking adults between the ages of 18 – 59 participated in the human challenge model.

Recruitment

Recruitment was through Liverpool School of Tropical Medicine by Ferreira's clinical team at LSTM.

ChIP-seq

Data deposition

☐ Confirm that both raw and final processed data have been deposited in a public database such as GEO.

☐ Confirm that you have deposited or provided access to graph files (e.g. BED files) for the called peaks.

Data access links
May remain private before publication.

For "Initial submission" or "Revised version" documents, provide reviewer access links. For your "Final submission" document, provide a link to the deposited data.

Files in database submission

Provide a list of all files available in the database submission.

Genome browser session
(e.g. UCSC)

Provide a link to an anonymized genome browser session for "Initial submission" and "Revised version" documents only, to enable peer review. Write "no longer applicable" for "Final submission" documents.

Methodology

Replicates

Describe the experimental replicates, specifying number, type and replicate agreement.

Sequencing depth

Describe the sequencing depth for each experiment, providing the total number of reads, uniquely mapped reads, length of reads and whether they were paired- or single-end.

Antibodies

Describe the antibodies used for the ChIP-seq experiments; as applicable, provide supplier name, catalog number, clone name, and lot number.

Peak calling parameters	*Specify the command line program and parameters used for read mapping and peak calling, including the ChIP, control and index files used.*
Data quality	*Describe the methods used to ensure data quality in full detail, including how many peaks are at FDR 5% and above 5-fold enrichment.*
Software	*Describe the software used to collect and analyze the ChIP-seq data. For custom code that has been deposited into a community repository, provide accession details.*

Flow Cytometry

Plots

Confirm that:

☒ The axis labels state the marker and fluorochrome used (e.g. CD4-FITC).

☒ The axis scales are clearly visible. Include numbers along axes only for bottom left plot of group (a 'group' is an analysis of identical markers).

☒ All plots are contour plots with outliers or pseudocolor plots.

☒ A numerical value for number of cells or percentage (with statistics) is provided.

Methodology

Sample preparation	Human mucosal cells from the inferior turbinate were obtained by curettage using a plastic Rhino-probe and incubated in cold PBS++ (PBS supplemented with 5mM EDTA and 0.5% FCS). Cells were dislodged by pipetting and centrifuged at 440g for 5 mins at 4oC. Supernatant was removed, and cells resuspended in 25µl of PBS++ with Live/DeadTM Fixable stain. After 15 minutes incubation on ice, an antibody cocktail to stain for epithelial surface marker expression was added and incubated for another 15 minutes. Samples were vortexed, resuspended in 3.5mls of PBS++ and filtered over a pre-wetted 70µm filter. Samples were transferred to a 5ml FACS tube, centrifuged and resuspended in 200µl Cell Fix.<br><br>For the in vitro analysis, confluent monolayers of Detroit 562 cells on 6 well plates were incubated with S. pneumoniae for 6 hours in 1% FCS phenol free alpha MEM (base media, Life Technologies). Cells were washed three times in PBS and gently lifted from the plate using a cell scraper in 300µl of base media supplemented with 1mM EDTA. Samples were transferred to 5ml FACS tubes and placed on ice for the duration of the protocol. Each cell sample was incubated with an antibody cocktail for epithelial surface marker expression (see Supplemental Information) for 30 minutes before rinsing in 1ml base media and centrifuging at 300g for 5 minutes at 4oC. Cells were fixed in 600µl of 4% PFA and acquired on a LSR II Flow Cytometer (BD Biosciences). Compensation was run and applied for each experimental replicate and voltages consistent throughout. Isotype controls (BD Biosciences), FL-1 and single stains were also run for each experiment. Samples were acquired until 300,000 events had been collected. Analyses were performed using FlowJo LLC version 10 software.
Instrument	Samples were acquired on LSRII Flow Cytometer (BD Biosciences).
Software	Analyses of data was performed using FlowJo LLC version 10 software.
Cell population abundance	In vivo: The entire sample was acquired as the starting population size varied between volunteers. Data was performed on the gated epithelial cell population and only samples containing 500 or more cells are reported. In vitro: Samples were acquired until 300,000 events had been collected.
Gating strategy	In vivo: Samples were gated into 'all cells', 'single cells', and finally 'EpCAM positive' cells, against an empty channel (AF-700-A) to analyse the epithelial cell population of the samples. In vitro: Cells collected from Detroit 562 confluent monolayers were gated into 'all cells', the population further defined to 'single cells', and finally exclusion of dead cells (defined by treatment with H2O2), lead to the live cell population used for analyses.

☒ Tick this box to confirm that a figure exemplifying the gating strategy is provided in the Supplementary Information.

Magnetic resonance imaging

Experimental design

Design type	*Indicate task or resting state; event-related or block design.*
Design specifications	*Specify the number of blocks, trials or experimental units per session and/or subject, and specify the length of each trial or block (if trials are blocked) and interval between trials.*
Behavioral performance measures	*State number and/or type of variables recorded (e.g. correct button press, response time) and what statistics were used to establish that the subjects were performing the task as expected (e.g. mean, range, and/or standard deviation across subjects).*

Acquisition

Imaging type(s) — *Specify: functional, structural, diffusion, perfusion.*

Field strength — *Specify in Tesla*

Sequence & imaging parameters — *Specify the pulse sequence type (gradient echo, spin echo, etc.), imaging type (EPI, spiral, etc.), field of view, matrix size, slice thickness, orientation and TE/TR/flip angle.*

Area of acquisition — *State whether a whole brain scan was used OR define the area of acquisition, describing how the region was determined.*

Diffusion MRI ☐ Used ☐ Not used

Preprocessing

Preprocessing software — *Provide detail on software version and revision number and on specific parameters (model/functions, brain extraction, segmentation, smoothing kernel size, etc.).*

Normalization — *If data were normalized/standardized, describe the approach(es): specify linear or non-linear and define image types used for transformation OR indicate that data were not normalized and explain rationale for lack of normalization.*

Normalization template — *Describe the template used for normalization/transformation, specifying subject space or group standardized space (e.g. original Talairach, MNI305, ICBM152) OR indicate that the data were not normalized.*

Noise and artifact removal — *Describe your procedure(s) for artifact and structured noise removal, specifying motion parameters, tissue signals and physiological signals (heart rate, respiration).*

Volume censoring — *Define your software and/or method and criteria for volume censoring, and state the extent of such censoring.*

Statistical modeling & inference

Model type and settings — *Specify type (mass univariate, multivariate, RSA, predictive, etc.) and describe essential details of the model at the first and second levels (e.g. fixed, random or mixed effects; drift or auto-correlation).*

Effect(s) tested — *Define precise effect in terms of the task or stimulus conditions instead of psychological concepts and indicate whether ANOVA or factorial designs were used.*

Specify type of analysis: ☐ Whole brain ☐ ROI-based ☐ Both

Statistic type for inference
(See Eklund et al. 2016) — *Specify voxel-wise or cluster-wise and report all relevant parameters for cluster-wise methods.*

Correction — *Describe the type of correction and how it is obtained for multiple comparisons (e.g. FWE, FDR, permutation or Monte Carlo).*

Models & analysis

n/a	Involved in the study
☐	☐ Functional and/or effective connectivity
☐	☐ Graph analysis
☐	☐ Multivariate modeling or predictive analysis

Functional and/or effective connectivity — *Report the measures of dependence used and the model details (e.g. Pearson correlation, partial correlation, mutual information).*

Graph analysis — *Report the dependent variable and connectivity measure, specifying weighted graph or binarized graph, subject- or group-level, and the global and/or node summaries used (e.g. clustering coefficient, efficiency, etc.).*

Multivariate modeling and predictive analysis — *Specify independent variables, features extraction and dimension reduction, model, training and evaluation metrics.*
